# Gut Bacterial Communities in the Ground Beetle *Carabus convexus*

**DOI:** 10.3390/insects15080612

**Published:** 2024-08-14

**Authors:** Tibor Magura, Szabolcs Mizser, Roland Horváth, Mária Tóth, Ferenc Sándor Kozma, János Kádas, Gábor L. Lövei

**Affiliations:** 1 Department of Ecology, Faculty of Science and Technology, University of Debrecen, Egyetem Sq. 1, H-4032 Debrecen, Hungary; mizser.szabolcs@science.unideb.hu (S.M.); horvath.roland@science.unideb.hu (R.H.); toth.maria@science.unideb.hu (M.T.); 2 HUN-REN–UD Anthropocene Ecology Research Group, University of Debrecen, Egyetem Sq. 1, H-4032 Debrecen, Hungary; kozmafs@gmail.com (F.S.K.); gabor.lovei@agro.au.dk (G.L.L.); 3 UD-GenoMed Medical Genomic Technologies Ltd., Clinical Centre, University of Debrecen, H-4032 Debrecen, Hungary; kadas.janos@ud-genomed.hu; 4 Flakkebjerg Research Centre, Department of Agroecology, Aarhus University, DK-4200 Slagelse, Denmark

**Keywords:** carabid, digestive tract, intestinal tract, microbiome, microorganisms, mutualism, operational taxonomic units, predators, symbionts

## Abstract

**Simple Summary:**

In symbiotic interactions, microorganisms associated with the intestinal tract, constituting the gut microbiome, are especially important, because they influence the life history and fitness of their host organisms. The gut microbiome of ground beetles, one of the most species-rich animal families, is poorly known, and research on sex-specific differences is almost completely missing. Investigating the gut bacterial microbiome of a widespread European carnivorous species, *Carabus convexus*, using next-generation high-throughput sequencing, we found 1138 different operational taxonomic units belonging to 21 bacterial phyla, 90 families, and 197 genera. One of the most abundant bacterial families and genera was previously not known from the digestive tracts of other ground beetle species. We also detected carbohydrate-degrading gut bacterial symbionts, which indicated possible plant feeding in this predatory species. Although the composition of the gut bacterial microbiome was not significantly different between the sexes, the gut symbionts showed more between-individual variability in females. This difference may result from the different mobility of sexes and the difference in reproductive investment.

**Abstract:**

Biological interactions, including symbiotic ones, have vital roles in ecological and evolutionary processes. Microbial symbionts in the intestinal tracts, known as the gut microbiome, are especially important because they can fundamentally influence the life history, fitness, and competitiveness of their hosts. Studies on the gut-resident microorganisms of wild animals focus mainly on vertebrates, and studies on species-rich invertebrate taxa, such as ground beetles, are sparse. In fact, even among the species-rich genus *Carabus*, only the gut microbiome of two Asian species was studied, while results on European species are completely missing. Here, we investigated the gut bacterial microbiome of a widespread European *Carabus* species, targeting the V3 and V4 regions of the 16S ribosomal RNA genes by next-generation high-throughput sequencing. We identified 1138 different operational taxonomic units assigned to 21 bacterial phyla, 90 families, and 197 genera. Members of the carbohydrate-degrading Prevotellaceae family, previously not detected in ground beetles, were the most abundant in the gut microbiome of the carnivorous *C. convexus*. Presumably, individuals from the studied wild populations also consume plant materials, especially fruits, and these carbohydrate-degrading bacterial symbionts can facilitate both the consumption and the digestion of these supplementary foods.

## 1. Introduction

Biotic interactions are widely recognized as fundamental drivers of ecological and evolutionary processes. Symbiotic relationships between microorganisms and multicellular organisms are particularly important, as these resident microorganisms, known as the microbiome, can fundamentally shape the life history, fitness, as well as ecological and evolutionary competitiveness of their hosts [1]. Endosymbiotic microorganisms, particularly those residing in digestive tracts, have garnered special attention due to their crucial roles in various biological processes [2]. The gut microbiome of humans [3] and some laboratory model organisms is particularly well studied [4,5]. In contrast, the gut microorganisms of wild animals have been less investigated, but this is quickly changing thanks to new sequencing methods and the gradually decreasing costs. Despite this, the majority of gut microbiome studies on wild animals have been conducted with mammals (e.g., [6,7,8,9,10]).

Recently, studies on the gut microbiome of insects have become a major interest, as gut microsymbionts play crucial roles in many biological processes, such as digesting plant fibers [11], production of essential vitamins and microelements [12], regulation of the hormonal [13] and the immune system [14], as well as the homeostasis of the host [15], degradation of toxins [16], and protection against pathogens and parasites [17]. A catalogue of the insect-associated gut bacteria of 218 species shows that Proteobacteria and Firmicutes make up 62.1 and 20.7% of the total reads, respectively [18].

In spite of this, our knowledge on the insect microbiome is fragmentary. Studies on the gut microbiome of ground beetles (Coleoptera: Carabidae), one of the most species-rich beetle families with more than 40,000 described species [19], are very limited and several studies are based on lab strains. To date, the gut microorganisms of only 40 species of ground beetles have been studied [20,21,22,23,24,25,26,27,28,29,30], but in 28 of them, no quantitative data are published about the OTUs or ASVs identified (Table 1). These include the following species: *Brachinus elongatulus* Chaudoir, 1876 [20], *Anisodactylus signatus* (Panzer, 1796) [24], *A. similis* LeConte, 1851 [20], *A. tricuspidatus* A. Morawitz, 1863 [24], *Chlaenius abstersus* Bates, 1873 [24], *C. naeviger* A. Morawitz, 1862 [24], *C. posticalis* (Fabricius, 1798) [24], *C. virgulifer* Chaudoir, 1876 [24], *Harpalus eous* Tschitscherine, 1901 [24], *H. griseus* (Panzer, 1796) [24], *H. jureceki* (Jedlicka, 1928) [24], *H. niigatanus* Schauberger, 1929 [24], *H. sinicus* Hope, 1845 [24], *Lachnolebia cribricollis* (A. Morawitz, 1862) [24], *Synuchus melantho* (Bates, 1883) [24], *S. nitidus* (Motschulsky, 1862) [24], *Dolichus halensis* (Schaller, 1783) [24], *Amara chalcites* Dejean, 1828 [24], *A. congrua* A. Morawitz, 1862 [24], *A. simplicidens* A. Morawitz, 1863 [24], *Myas coreana* (Tschitscherine, 1895) [24], *Pterostichus fuligineus* A. Morawitz, 1862 [24], *P. microcephalus* (Motschulsky, 1861) [24], *P. microcephalus* (Motschulsky, 1861) [24], *P. planicollis* (Motschulsky, 1861) [24], *P. samurai* (Lutshnik, 1916) [24], *P. serripes* (LeConte, 1875) [20], *P. sulcitarsis* A. Morawitz, 1863 [24], *Carabus (Lepto-carabus) arboreus* Lewis, 1882 [24], and *C. albrechti* A. Morawitz, 1862 [24]. In these 40 species, a wide range of microorganisms were detected, ranging from 2 OTUs (in *Anisodactylus santaecrucis*) to 2647 OTUs (in 30 individuals of *Pterostichus melas*).

Within the Carabinae subfamily, endosymbiotic microorganisms have only been explored in two species, *Carabus (Leptocarabus) arboreus* Lewis, 1882 and *C. albrechti* A. Morawitz, 1862 [24]. Both studied *Carabus* species are native to Asia, and for the sequencing of the gut microbiome, the individuals were collected in northern Japan [24]. To our knowledge, there is only one study on the gut microbiome of European *Carabus* species. However, the evaluation in this study was based on ASVs, and due to the great lack of reference data, the bacterial community could only imperfectly be described: At the phylum level, 43% of the detected taxa were not present in the database. At the family and genus levels, 63% and 77% were unidentified, respectively [31]. Thus, for comparative purposes, an OTU-based analysis is needed on European *Carabus* species.

Gut microbiomes may differ between the sexes to differences in mobility [18]. There are documented differences in mobility and, consequently, in the home range between ground beetle males and females, especially during the reproductive period [19]. Therefore, male beetles may acquire a more diverse set of microbial symbionts from their environment than females, resulting in sex-specific differences in the gut microbiome. Still, to date, only one study has investigated such sex-specific differences in ground beetles [21].

To address the above-mentioned knowledge gaps, here, focusing on wild populations, we provide the first description of the gut bacterial microbiome of a widespread European *Carabus* species using next-generation high-throughput sequencing of the bacterial 16S rRNA gene. Specifically, we hypothesized that the more wide-ranging males would have a more varied microbiome than females.

## 2. Materials and Methods

### 2.1. Studied Species and Sampling Design

For this study, we chose the common carabid, *Carabus convexus* Fabricius, 1775. The distribution of this Western Palaearctic species includes much of Europe, Turkey, the Caucasus, western Siberia, and northern Kazakhstan [32]. In Central Europe, this eurytopic and meso-(thermo) philous species is mainly nocturnal and carnivorous with extraintestinal digestion. It reproduces in spring, with teneral adults emerging in late July to early August. Adults go to overwinter from November [32]. This medium-sized (14–20 (23) mm), brachypterous, dispersal-limited ground beetle is generally a forest-associated species [32,33].

*C. convexus* adults were collected in the Great Forest of Debrecen (47°34′36″ N; 21°37′43″ E). This protected forest is part of the Natura 2000 network (site code: HUHN20033), located near the second largest city in Hungary, Debrecen. In this lowland forest, four mature stands (>120 years) dominated by English oak (*Quercus robur*) were selected. All stands were at least 3 ha (3.71 ha, 3.75 ha, 3.94 ha, and 3.04 ha, respectively) and were at a mean distance of 396.5 m from each other. We operated unbaited live pitfall traps (15 traps at each site) during the main spring activity period from the end of March to mid-May in 2021. Pitfall traps were placed randomly but at least 10 m apart from each other to ensure statistical independence [34], as well as at least 50 m from forest edges to avoid edge effects [35]. Traps were plastic boxes (170 mm × 110 mm, 105 mm height). Shredded leaves inside the traps provided shelter to reduce intra- and interspecific predation. Fiberboard roofs (200 mm × 200 mm) protected the traps from rain and prevented bycatch and predation by birds and/or mammals. Traps were checked every 3 days. In the studied habitats, *C. convexus* is a dominant member of the ground-dwelling invertebrate assemblages. Trapped *C. convexus* adults (15 females and 16 males) were transported to the laboratory; others were released near the trap. In the laboratory, the beetles were sexed, euthanized by freezing, and stored individually in centrifuge tubes (2 mL) filled with 96% ethanol at 5 °C until dissection.

### 2.2. Gut Dissection, Microbial DNA Extraction, Amplification, and Sequencing

Stored beetles (15 females and 16 males) were dissected under a stereo microscope (Delta IPOS-810, Delta Optical, Nowe Osiny, Poland) within 3 days to avoid changes in the gut microbiome [24]. Before dissection, beetles were rinsed in 96% ethanol and then rinsed with sterile Ringer’s solution to avoid contamination. After the beetles were pinned to a plate, the legs and elytra were removed and flushed again with 96% ethanol and sterile Ringer’s solution. To access the intestinal tracts, tergites were cut lengthwise on both sides and removed. The foregut and midgut were carefully extracted, flushed with 96% ethanol, and placed in sterile centrifuge tubes (1.5 mL) filled with 96% ethanol and stored at −80 °C. After every dissection, tools used for dissections, as well as the plates were cleaned and sterilized with 96% ethanol.

DNA extraction was performed immediately after the end of the sampling period (mid-May). Before microbial DNA extraction, the gut samples were rinsed with ice-cold phosphate buffer (PBS 280–315 mOsm/kg, pH 7.4). Bacterial DNA was extracted using the QIAamp PowerFecal Pro DNA Kit (QIAGEN GmbH, Hilden, Germany), following the manufacturer’s guidelines. The concentration of the extracted DNAs was measured using a DeNovix spectrophotometer (DeNovix, Inc., Wilmington, DE, USA).

Widely used, robust universal primers for PCR amplification of the V3-V4 region of bacterial 16S ribosomal RNA (rRNA) genes were used (Sigma/Merck KGaA, Darmstadt, Germany) according to the Illumina 16S Metagenomic Library Preparation Guide (15044223-B). The primer sequences were as follows:

16S Amplicon PCR Forward Primer = 5′ TCGTCGGCAGCGTCAGATGTGTATAAGAGACAGCCTACGGGNGGCWGCAG.

16S Amplicon PCR Reverse Primer = 5′ GTCTCGTGGGCTCGGAGATGTGTATAAGAGACAGGACTACHVGGGTATCTAATCC (Sigma/Merck KGaA, Darmstadt, Germany). Microbial DNA in a 25 μL mixture (5 μL DNA, 2.5 μL of each primer (2 μM), 5 μL of PCR grade water, and 12.5 μL KAPA HiFi HotStart Ready Mix, KAPA/Roche Biosystems Ltd., Cape Town, South Africa) was amplified with the following reaction conditions: denaturation at 95 °C for 3 min; 25 cycles of 95 °C for 30 s, 55 °C for 30 s, and 72 °C for 30 s; and fragment elongation at 72 °C for 5 min. PCR products were purified using the Agencourt AMpure XP PCR purification system (Beckman Coulter, Brea, CA, USA). In the second stage of PCR, Dual Illumina indices (The Nextera XT Index Kit, Illumina, Inc., San Diego, CA, USA) were added to the PCR products in a 50 μL mixture (5 μL purified amplicon PCR product, 25 μL KAPA HiFi Hot-Start ReadyMix, 5 μL of each index primer, and 10 μL of PCR grade water) using denaturation at 95 °C for 3 min; 8 cycles of 95 °C for 30 s, 55 °C for 30 s, 72 °C for 30 s; and a final extension at 72 °C for 5 min. Again, the amplified fragments were purified using the Agencourt AMpure XP PCR purification system. The quality and yield of 16S rRNA V3−V4 libraries were assessed using Agilent DNA 1000 Kit on Agilent 2100 BioAnalyzer (Agilent Technologies, Santa Clara, CA, USA).

Library samples were paired-end sequenced on the Illumina MiSeq platform (Illumina, Inc., San Diego, CA, USA) using the MiSeq Reagent Kit v3 with 600 cycles, producing on average 300,000 raw reads per sample. During the analyses, the recommendations by Eisenhofer et al. [36] were followed. Therefore, negative controls were also used: DNA extraction control (sterile water instead of gut sample during the DNA extraction) and PCR amplification control (sterile water instead of template DNA during PCR amplification). Negative controls had no microbial reads, proving the robustness of the procedure.

### 2.3. Bioinformatic and Statistical Analyses

Forward and reverse paired-end reads were processed using the FROGS pipeline [37]. Paired-end reads were merged using the FLASH tool [38], followed by quality control for primer-filtered reads using the VSEARCH [39]. Chimeras were detected and removed using the VSEARCH by the de novo UCHIME method [39,40]. Dereplicated, quality-controlled sequences were clustered into operational taxonomic units (OTUs) by the VSEARCH using the identity threshold of 97% [39]. OTUs below 0.005% abundance were considered noise and eliminated [41]. The taxonomic assignment of OTUs was performed using the BLAST [42] on the SILVA 16S database for bacterial sequences [43].

All statistical analyses were made in the R program environment (version 4.3.1, [44]). The bioinformatically processed raw read counts were median normalized using the *phyloseq* package (v. 1.30.0) [45] to control the differences in library size (i.e., sequencing depth) [46]. The diversity in identified bacterial genera was evaluated by the richness of genera, the Shannon–Wiener index, the evenness, and the dominance indices using the *vegan* package (v. 2.6-4) [47]. The composition of the gut bacterial communities in female and male beetles was displayed by non-metric multidimensional scaling (NMDS) ordination using the Bray–Curtis index of dissimilarity calculated using the *vegan* package [47]. Convex hulls around the gut bacterial samples were shown using the standard errors of the averages of samples multiplied by the 95% confidence value from the Chi-squared distribution (with 2 df) with the help of the *vegan* [47] and *MASS* (v. 7.3.60) [48] packages. Also, the *vegan* package [47] was used to show significant differences in averages (centroids).

## 3. Results

### 3.1. Sequencing Data

All the collected beetles had unworn mandibles that indicated overwintered individuals in their first breeding season. The sequencing yielded a total of 1138 OTUs (Table S1; mean number of OTUs ± SD per sample: 355.42 ± 57.22, with a total read count of 5,140,946, and a mean of 165,837 reads ± 69,654.33 (S.D.)). Metadata and raw sequence reads are deposited in the NCBI SRA database (BioProject PRJNA1051584, https://www.ncbi.nlm.nih.gov/bioproject/PRJNA1051584, accessed on 12 December 2023).

### 3.2. Diversity and Composition of the Gut Bacterial Communities

**Figure 1:**
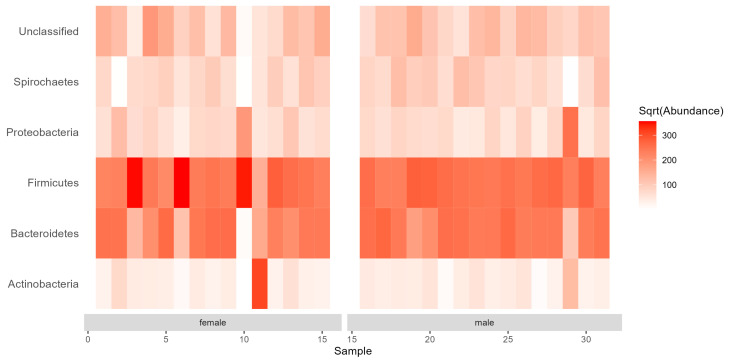
Abundance (median normalized read counts) of the bacterial phyla with >1% relative abundance in gut samples of *Carabus convexus* females (n = 15) and males (n = 16).

We found a total of 21 bacterial phyla. The most common (>5% of the relative read counts in all samples) phyla were as follows: Firmicutes (42.95% of the total read counts, a bit higher in females (44.64%) than in males (41.37%)), Bacteroidetes (33.31% in total, 30.36% in females, 36.07% in males), an unclassified phylum (8.36% in total, 8.84% in females, 7.90% in males), and Proteobacteria (5.16% in total, 4.98% in females, 5.33% in males) (Figure 1). These four phyla represented 89.78% of the total read counts. One bacterial family (Armatimonadetes) was found only in female beetles, at a very low relative abundance (0.05%).

**Figure 2:**
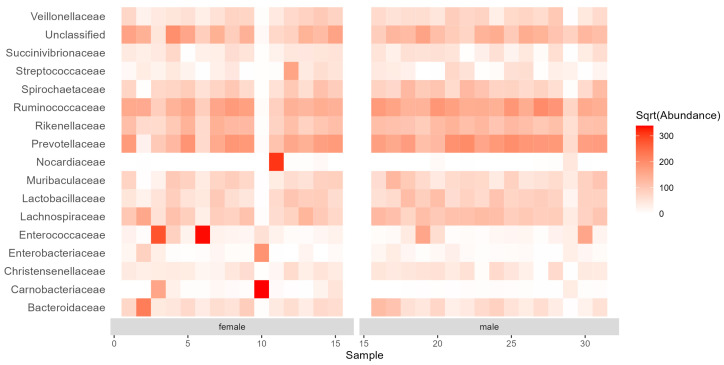
Abundance (median normalized read counts) of the bacterial families common (>1% relative abundance in all samples) in gut samples of *Carabus convexus* females (n = 15) and males (n = 16).

A total of 90 bacterial families were identified. Based on the relative read counts, the most abundant (>5% of the relative read counts; Figure 2) ones included: Prevotellaceae (15.70% in total, 13.08% in females, 18.17% in males), Ruminococcaceae (14.04% in total, 11.76% in females, 16.17% in males), an unclassified family (8.36% in total, 8.84% in females, 7.90% in males), Rikenellaceae (7.45% in total, 6.73% in females, 8.14% in males), Lachnospiraceae (6.05% in total, 5.49% in females, 6.58% in males), and Enterococcaceae (5.44% in total, 8.78% in females, 2.32% in males). These six families accounted for 57.05% of the total read counts. Three families (Peptococcaceae, Mycoplasmataceae, and Spiroplasmataceae) were found only in female guts but at a low relative abundance (0.05%, 0.02%, and 0.01% of the reads, respectively). Similarly, three families (Helicobacteraceae, Halomonadaceae, and Neisseriaceae) with very low relative abundances (0.03%, 0.02%, and 0.00004% of the reads, respectively) were present only in males.

**Figure 3:**
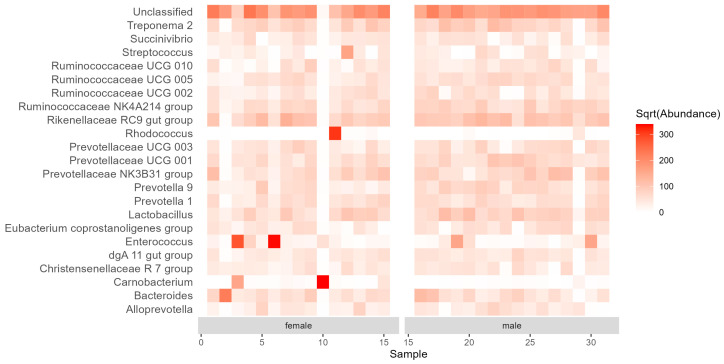
Abundance (median normalized read counts) of the bacterial genera common (>1% relative abundance in all samples) in *Carabus convexus* female (n = 15) and male (n = 16) guts.

At the genus level, a total of 197 bacterial genera were identified. The most abundant (>5%) genera (Figure 3) belonged to an unclassified genus (20.12% in total, 19.45% in females, 20.75% in males), the RC9 genus group from Rikenellaceae (5.85% in total, 5.17% in females, 6.49% in males), and *Enterococcus* (5.40% in total, 8.71% in females, 2.30% in males). These three genera made up 31.37% of the total reads. Ten occasional bacterial genera were found only in males, while another 10 were only in females (all of them with ≤ 0.06% of read counts).

The richness of bacterial genera did not differ significantly between the sexes; however, the Shannon–Wiener index and the evenness index were significantly higher, while the dominance index was significantly lower in males compared to females (Table S2).

**Figure 4:**
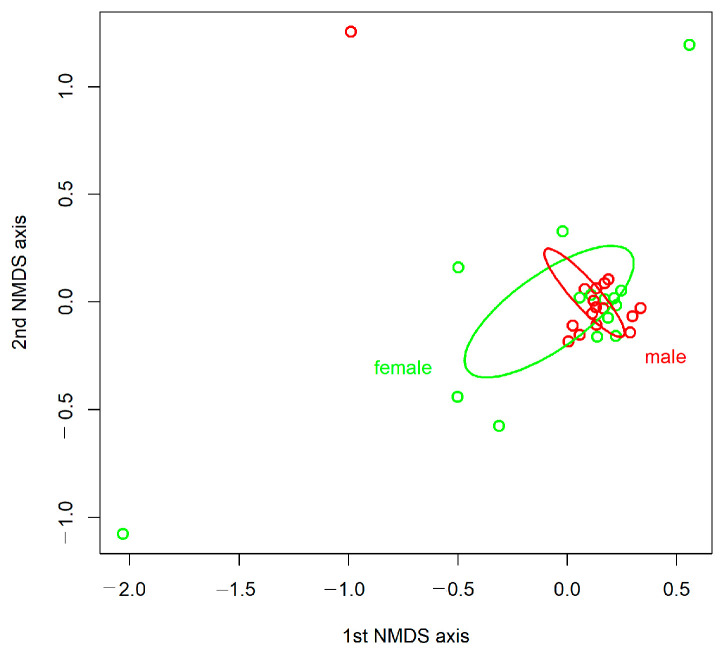
Ordination (non-metric multidimensional scaling, NMDS) of the bacterial operational taxonomic units (OTUs) from gut samples of *Carabus convexus* female and male adults using the Bray–Curtis index of dissimilarity (open green circles represent gut bacterial samples from female beetles, n = 15, while open red circles from male beetles, n = 16). Convex hulls (ellipses) around the samples from female and male individuals are based on the product of the standard errors of the averages of samples and the 95% confidence values. Final stress value: 12.0791.

Although there were differences in the relative abundances between females and males at all taxonomic levels (phylum, family, genus; Figure 1, Figure 2 and Figure 3), the NMDS ordination using the Bray–Curtis index of dissimilarity (Figure 4) did not show significant differences between the sexes (R^2^ = 0.022, *p* = 0.635, number of permutations: 999). However, β-diversity (expressed by the convex hull volume in the ordination space) of the gut bacterial communities in female beetles was higher compared to male ones (Figure 4).

## 4. Discussion

All previous studies on the gut bacterial microbiome of ground beetles focused on the Brachininae, Harpalinae, Platyninae, Pterostichinae, and Trechinae subfamilies [20,21,22,23,24,25,26,27,28,29,30]. Studies on other subfamilies, including Carabinae, are almost entirely lacking (but see Kudo et al. [24], and Magura et al. [31]). Here, using next-generation high-throughput 16S amplicon sequencing and analyzing OTUs, we provided the first gut bacterial microbiome analysis of a Eurasian member of the Carabinae subfamily. But note that there are limitations of 16S rRNA gene-based analysis [36].

Our sequencing data revealed a total of 1138 different OTUs. This number is roughly similar to the 1245 OTUs detected from gut samples of 32 *C. pallipes* males [25]. In *C. convexus*, the mean number of OTUs was 355.42 per beetle. Approximately similar numbers (108–195 OTUs per beetle) are present in the intestinal tracts of 39 individuals of four *Bembidion* species (*B. decorum*, *B. modestum*, *B. punctulatum*, and *B. varicolor* [30]). Considerably, fewer OTUs were identified when terminal restriction fragment length polymorphism of polymerase chain reaction-amplified 16S rRNA genes was used [23,26,28]. Sometimes even using amplicon sequence variants (ASVs) does not yield more microbial species [21]. All of the above indicate the potential incompleteness of reference data. Furthermore, the diversity in the gut bacterial microbiome of various insect taxa can also vary. Some insects (e.g., honeybees, reed beetles, and fruit flies) host fewer than 10 species/OTUs in their guts [12], while others (e.g., cockroaches and termites) often harbor > 1000 OTUs [12,49]. Among beetles, ground beetles host diverse gut bacterial communities [30] with significant differences in the composition of the microbiome between different feeding groups [24,50]. Generally, omnivorous beetles have higher gut bacterial diversity than carnivorous and herbivorous ones [18].

The 21 bacterial phyla identified are comparable to the total of 15 phyla in *P. rufipes* gut samples [27]. Similar to previous studies [18,50], the major groups in *C. convexus* were Firmicutes, Bacteroidetes, and Proteobacteria making up 81.42% of the gut bacterial community. Similarly, bacteria from the Firmicutes phylum were the most dominant in the guts of several carnivorous ground beetles, including *B. elongatulus* [20,21], *C. pallipes* [22,25], and *P. jessoensis* [22], suggesting that Firmicutes are the most prominent symbiotic bacteria in carnivorous beetle guts [25]. However, in other predatory ground beetles (*B. decorum*, *B. modestum*, *B. punctulatum*, *B. varicolor* [30], *P. chalcites* [28], *P. melas* [29], and *P. serripes* [20]), as well as in the omnivorous *Amara similis* [20], Proteobacteria is the dominant phylum. In the omnivorous *P. rufipes*, the most common phylum is Tenericutes [27]. These results underline that apart from the diet, taxonomy also influences the composition of the gut bacterial community [50].

Of the 90 bacterial families identified in the gut samples of *C. convexus* individuals, Prevotellaceae (15.70%), Ruminococcaceae (14.04%), Rikenellaceae (7.45%), Lachnospiraceae (6.05%), and Enterococcaceae (5.44%) were the most common. The study on *C. albrechti* and *C. arboreus* from northern Japan also revealed that Enterococcaceae are among the most common taxa in the microbiome [24]. Enterococcaceae are the most abundant in various herbivorous, omnivorous, and carnivorous species [22,24,25,29], although not in *P. chalcites* [28], *B. punctulatum* [30], or *P. rufipes* [27]. The protein-digesting Rikenellaceae are present in carnivorous species [28]. The plant material-degrading Lachnospiraceae and Ruminococcaceae are also present in the carnivorous *P. chalcites* [28] and several *Bembidion* species [30]. However, Prevotellaceae, the most abundant family in the gut microbiome of *C. convexus*, has not previously been described in other ground beetles. Prevotellaceae are present in the guts of other phytophagous insects, such as *Agrilus mali* Matsumura, 1924 [51], *Dendroctonus rhizophagus* Thomas and Bright, 1970 [52], and *Nilaparvata lugens* (Stal, 1854) [53]. The presence of carbohydrate-degrading Prevotellaceae in wild *C. convexus* suggested that *C. convexus* may really consume plant materials, probably fruits [54]. Thus, all the above-mentioned plant material-degrading gastrointestinal bacterial inhabitants in *C. convexus* can facilitate the consumption and digestion of plant material [24,55], even influencing the ecological role of the host species in the trophic network [29]. Alternatively, the presence of carbohydrate-degrading bacterial symbionts may be secondary, i.e., it may get into the predator beetle’s gut via its herbivorous prey. However, this is unlikely for *C. convexus* where the major prey, earthworms, are not herbivores.

At the genus level, there were twice as many unclassified OTUs as at the phylum or family levels, indicating that taxa present in the studied gut samples may not have yet been included in the reference database. Nevertheless, members of a genus from the Rikenellaceae family and the *Enterococcus* genus were abundant symbionts in the guts of *C. convexus*. Bacteria belonging to the *Enterococcus* genus are common members of the insect gut microbiome [18,30,56]. They usually occur in herbivorous insects and are also prominent members of the forest litter and topsoil layer, so they can easily enter the intestinal tracts of forest-associated ground-dwelling carnivorous beetles [56]. *Enterococcus* spp. play a key role in, among others, metabolic adaptability against pathogenic and plant toxins [57], but they are also facultative pathogens [30]. Previous studies also confirmed the dominance of *Enterococcus* spp. in the gut of both omnivorous [20,26] and carnivorous ground beetles [20,21,22,25,27,28,29,30].

The gut microbiome may differ between the sexes [18]. For example, bacterial symbionts can cause sex-specific reproductive incompatibilities, influencing host reproduction and fitness (the so-called male-killing bacteria). Other gut inhabitants (e.g., *Klebsiella oxytoca*) can also increase the sexual competitiveness of males and enhance their survival, as in the Mediterranean fruit fly, *Ceratitis capitata* (Wiedemann, 1824) [12]. Still, only a single previous study on ground beetles investigated such sex-specific differences, finding no significant differences between females and males [21]. Similar to our hypothesis, the Shannon–Wiener index and the evenness index of bacterial genera were significantly higher in males than in females (Table S2), suggesting that during the reproductive period, more active male beetles may acquire a more diverse set of microbial symbionts from their environment than females. Contrary to our hypothesis, we found a higher β-diversity (expressed by the convex hull volume in the ordination space, Figure 4) of the gut bacterial communities in female *C. convexus* adults compared to male ones. This difference may be because females, to supply a huge amount of energy to produce and ripen eggs, cover a larger range to find high-quality food items [19]. Furthermore, females may try to lay their eggs far away from each other, in order to avoid cannibalism among the hatching larvae [19]. Thus, stochastically acquired bacteria from various food items and different microenvironments [58] may contribute to the detected, higher between-individual microbiome variability in female beetles.

Our study on the gut bacterial microbiome of a European *Carabus* species was based on individuals from wild populations of a relatively small geographical region. As the diversity and composition of the gut bacterial associates can be profoundly influenced by the host environments [12,24,31], studying other populations from other European localities, and even from different latitudes and/or habitat types, as well as focusing on other *Carabus* species, can considerably contribute to a detailed understanding of the gut bacterial microbiome of European *Carabus* species. Furthermore, exploring diet-derived modifications of the host microbiome could shed light on how diet influences the gut microbiome composition in *C. convexus*. The small geographical range of our collection is a good start, but future studies could systematically investigate diet-related variations in both laboratory and field settings. Additionally, investigating gut microbiome abundance and composition differences between generations could reveal if there are obligate symbionts in *C. convexus* and shed light on the transmission modes of these symbionts—whether vertical, horizontal, or a combination thereof.

Recently, there has been a growing interest in the sensitivity of the microbiome to environmental disturbances (e.g., exposure to pesticides [27,29] and antibiotics [28]; or urbanization-related environmental disturbance [31]). The results consistently show that symbiont microorganisms are valuable tools to evaluate symbiotoxicity [59]. There is also an emerging shift from studying individual organisms towards symbiotic interactions (e.g., for pathogens [60,61]). Symbiotic organisms are key elements in ecosystem processes [62]; therefore, studies on symbiotic interactions can usefully contribute to plan and implementing management tools that ensure continued ecosystem functioning.

## Figures and Tables

**Table 1:** Ground beetle subfamilies and species whose gut microbiome have been studied, their feeding habit, and the number of microorganisms (operational taxonomic units, OTUs and amplicon sequence variants, ASVs) detected.

Subfamily	Species	Feeding Habit	Location	No. of Individuals Analyzed	No. of OTUs/ASVs	Reference
Brachininae	*Brachinus elongatulus* Chaudoir, 1876	Carnivore	USA	11	37 ASVs/gut (mean)	[21]
	*Pheropsophus jessoensis* A. Morawitz, 1862	Carnivore	Republic of Korea	10	~100–300 ASVs/ gut	[22]
Harpalinae	*Anisodactylus sanctaecrucis* (Fabricius, 1798)	Omnivore	USA	6	2 OTUs	[23]
	*Chlaenius pallipes* (Gebler, 1823)	Carnivore	South Korea	8	~110–190 ASVs/gut (mean)	[22]
	*C. pallipes* (Gebler, 1823)	Carnivore	South Korea	32	~25–50 OTUs/ gut (mean)	[25]
	*Harpalus pensylvanicus* (DeGeer, 1774)	Omnivore	USA	4	6 OTUs	[23]
	*H. pensylvanicus* (DeGeer, 1774)	Omnivore	USA	80	35 OTUs (total)	[26]
	*Pseudoophonus rufipes* (DeGeer, 1774)	Omnivore	Italy	29	798 OTUs (total)	[27]
Pterostichinae	*Poecilus chalcites* (Say, 1823)	Carnivore	USA	15	19 OTUs (total)	[28]
	*Pterostichus melas italicus* (Dejean, 1828)	Carnivore	Italy	30	2647 OTUs (total)	[29]
Trechinae	*Bembidion decorum* (Panzer, 1799)	Carnivore	Poland	9	195 OTUs/gut (mean)	[30]
	*B. modestum* (Fabricius, 1801)	Carnivore	Poland	10	108 OTUs/gut (mean)	[30]
	*B. punctulatum* Drapiez, 1820	Carnivore	Poland	10	164 OTUs/gut (mean)	[30]
	*B. varicolor* (Fabricius, 1803)	Carnivore	Poland	10	165 OTUs/gut (mean)	[30]

## Data Availability

The data used in this study are available in the Supplementary Material, Table S1.

## Supplementary Materials

The following supporting information can be downloaded at https://www.mdpi.com/article/10.3390/insects15080612/s1, Table S1: Median normalized number of reads and taxonomic hierarchy of the 1138 different bacterial operational taxonomic units (OTUs) in gut samples of 15 female and 16 male *Carabus convexus* beetles. Table S2: Average diversity values (±SD) of the identified bacterial genera in gut samples of 15 female and 16 male *Carabus convexus* beetles. Different letters indicate significant differences in diversity measures between sexes by one-way analysis of variance.
