# Supplementary material for: Gut Bacterial Communities in the Ground Beetle Carabus convexus
Source: Insects. 2024 Aug 14;15(8):612. doi: 10.3390/insects15080612 (PMC11354712; doi:10.3390/insects15080612)
Supplement: Supplementary file 1 [file insects-15-00612-s001.zip › TableS2.pdf]

Table S2. Average diversity values (± SD) of the identified bacterial genera in gut samples of 15 female and 16 male *Carabus convexus* beetles. Different letters indicate significant differences in diversity measures between sexes by one-way analysis of variance.

	Richness	Shannon–Wiener index	Evenness	Dominance index
Female beetles	95.47 ± 8.84[a]	2.88 ± 0.88[a]	0.63 ± 0.19[a]	0.17 ± 0.19[a]
Male beetles	98.38 ± 6.44[a]	3.45 ± 0.16[b]	0.75 ± 0.03[b]	0.05 ± 0.01[b]
